# Neural Underpinnings of Proactive Interference in Working Memory: Evidence From Patients With Unilateral Lesions

**DOI:** 10.3389/fneur.2021.607273

**Published:** 2021-02-10

**Authors:** Stephanie K. Ries, Krista L. Schendel, Timothy J. Herron, Nina F. Dronkers, Juliana V. Baldo, And U. Turken

**Affiliations:** ^1^School of Speech, Language, and Hearing Sciences, Center for Clinical and Cognitive Neuroscience, San Diego State University, San Diego, CA, United States; ^2^Veterans Affairs Northern California Health Care System, Martinez, CA, United States; ^3^Department of Psychology, University of California, Berkeley, Berkeley, CA, United States; ^4^National Research University Higher School of Economics, Neurolinguistics Laboratory, Moscow, Russia

**Keywords:** working memory, ventral white matter pathways, Inferior fronto-occipital fasciculus, proactive interference resolution, unilateral stroke-induced brain lesions, extreme/external capsule

## Abstract

Proactive interference in working memory refers to the fact that memory of past experiences can interfere with the ability to hold new information in working memory. The left inferior frontal gyrus (LIFG) has been proposed to play an important role in resolving proactive interference in working memory. However, the role of white matter pathways and other cortical regions has been less investigated. Here we investigated proactive interference in working memory using the Recent Probes Test (RPT) in 15 stroke patients with unilateral chronic lesions in left (*n* = 7) or right (*n* = 2) prefrontal cortex (PFC), or left temporal cortex (*n* = 6). We examined the impact of lesions in both gray and white matter regions on the size of the proactive interference effect. We found that patients with left PFC lesions performed worse overall, but the proactive interference effect in this patient group was comparable to that of patients with right PFC lesions, temporal lobe lesions, and controls. Interestingly, the size of the interference effect was significantly correlated with the degree of damage in the extreme/external capsule and marginally correlated with the degree of damage in the inferior frontal occipital fasciculus (IFOF). These findings suggests that ventral white matter pathways connecting the LIFG to left posterior regions play a role in resolving proactive interference in working memory. This effect was particularly evident in one patient with a very large interference effect (>3 SDs above controls) who had mostly spared LIFG, but virtually absent ventral white matter pathways (i.e., passing through the extreme/external capsules and IFOF). This case study further supports the idea that the role of the LIFG in resolving interference in working memory is dependent on connectivity with posterior regions via ventral white matter pathways.

## Introduction

Proactive interference in working memory refers to the fact that the memory of the past can interfere with the ability to hold new information in working memory. For a related example in our everyday lives, if you ride or drive to the same place every day and park your bike or car in the same spot, your memory of the spot you parked in on previous days can interfere with your memory of where you parked today (1). The ability to overcome proactive interference in working memory has been shown to decrease with age and has been associated with cognitive decline [e.g., (2)]. In addition, patients with frontal brain damage have been shown to have increased susceptibility to proactive interference in working memory [(3–5), see below].

**Figure 1:**
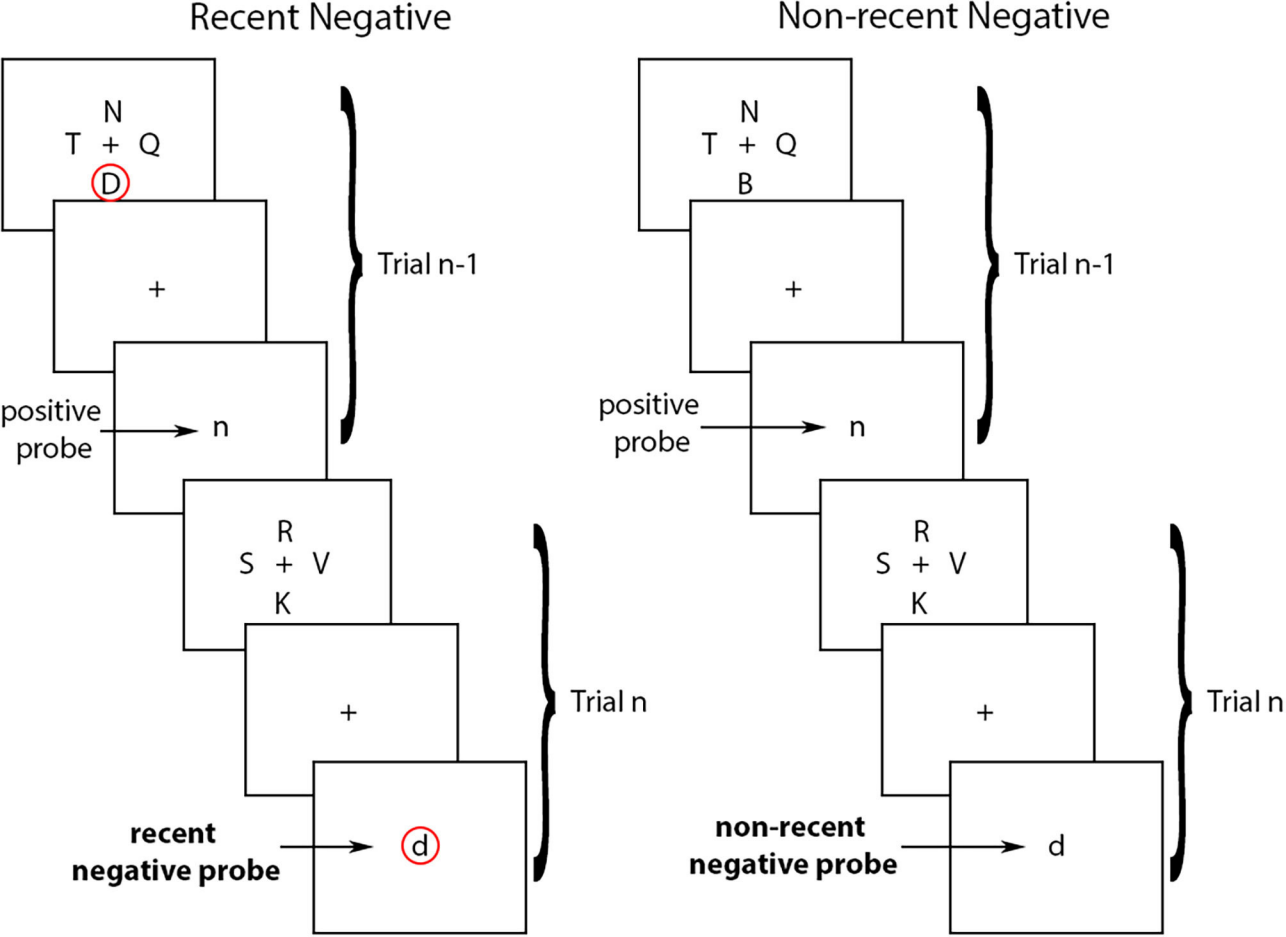
Recent negative and non-recent negative trials in the Recent Probes Test. In the recent negative trials, the probe at trial n is not part of the target-set on that trial but was part of the target-set at trial n-1 (circled in red). In the non-recent negative trials, the probe at trial n was not part of the target-set on trial n or on trial n-1. Arrows indicate the type of probes in each trial.

Experimentally, one of the most widely used tasks to study proactive interference in working memory is the Recent Probes Test (RPT) developed by Monsell (6) (see Figure 1). In the RPT task, participants see a set of stimuli (i.e., the target-set), and then a central stimulus (i.e., the probe). The goal of the task is to decide whether or not the central probe was part of the target-set by holding the items of the target-set in working memory. Typically, the stimuli are letters, but non-verbal stimuli have also generated similar effects [see (7) for a review]. The variable that is manipulated is whether or not the probe at trial *n* was part of the target-set presented in the preceding trial (trial *n*-1). If the probe at trial *n* is not part of the target-set on that trial but was part of the target-set at trial *n*-1, trial *n* is referred to as a recent negative (RN). If the probe at trial *n* was not part of the target-set on trial *n* or on trial *n*-1, trial *n* is referred to as a non-recent negative (NN). The proactive interference effect refers to the fact that performance is typically worse for RN than for NN trials due to proactive interference from the recently presented items (i.e., from the target-set from trial *n*-1, or the letter “D” in in the example in Figure 1). In other words, a recently presented item (e.g., from the previous trial) can interfere with working memory on a subsequent trial thereby reducing one's ability to correctly reject a non-target item.

The left prefrontal cortex (PFC) and in particular the left inferior frontal gyrus (LIFG) have been proposed to play a key role in proactive interference resolution in working memory [see (1) and (7) for reviews]. Using the RPT, proactive interference has been found to be associated with an increased BOLD signal in the left PFC (8, 9), and to a lesser extent in right PFC (10, 11). Moreover, the role of the LIFG in the resolution of proactive interference has been investigated in patients with stroke-induced focal lesions involving this region (3, 12). Patients with LIFG lesions, especially in Brodmann's Area 45, have been shown to have larger proactive interference effects in the RPT in comparison to both aged-matched controls and patients with frontal lesions that do not include the LIFG (12). Thus, the LIFG has been established as a cortical region that is associated with the ability to resolve proactive interference in working memory.

Additional brain regions beyond the PFC have been associated with other aspects of memory, such as formation, recollection, and maintenance of temporary memory traces. In particular, a large body of evidence indicates a critical role for bilateral medial temporal cortex, in particular the hippocampi, in memory formation and recollection [see e.g., (13, 14) for reviews]. The hippocampi have however not been commonly associated with working memory processes in particular even if, of relevance to proactive interference, the hippocampus has been shown to be active in situations requiring the disambiguation of overlapping memory sequences [e.g., (15)]. In addition, the ventral PFC has been shown to be active in concert with the medial and posterior hippocampus when inhibition of irrelevant stimuli is required (16). A recent study by Lorenc et al. (17) also found that the deliberate forgetting of unwanted information was associated with BOLD signal change in early visual cortex. This effect was in contrast to that observed for maintenance of currently relevant information, which engaged visual, parietal, and frontal regions (17). How these separate brain regions communicate to enable us to overcome proactive interference in working memory likely depends on interconnecting white matter pathways. To date, however, there is little known regarding the involvement of specific white matter pathways in the resolution of proactive interference.

Interestingly, proactive interference has also been suggested to be present in language when we are retrieving words from memory as we speak (18, 19). When several words of the same semantic category have to be produced, proactive interference can affect the selection of the target word (20–22). This semantic interference effect (due to residual activation of words in semantic memory) is analogous to the proactive interference effect that has been observed in the RPT. In both instances, it is the residual activation of recent or familiar representations in working memory that causes a decrease in performance when attempting to reach a decision on a subsequent trial (i.e., trial n). It is thus highly probable that similar neurobiological mechanisms may be involved in resolving proactive interference in both the visual and language domains. Consistent with this, fMRI and lesion studies have likewise implicated the LIFG as playing an important role in proactive interference resolution in language production (23–25). Patients with LIFG lesions, for example, have been shown to have larger semantic interference effects in picture naming compared to controls (24, 25). This is also the case when comparing patients with LIFG lesions to those with right PFC lesions (19).

In addition, patients with stroke-induced lesions to the left MTG and STG also show increased semantic interference effects (25). This finding is consistent with the idea that the left posterior middle temporal gyrus (MTG) is thought to play a central role in the activation of lexical representations in language production and comprehension [e.g., (26, 27)]. In particular, temporal regions, such as the left middle and posterior sections of the MTG and also the posterior superior temporal gyrus (STG) show increased BOLD signal in semantically-related compared to unrelated contexts in language production (25, 28). It is therefore reasonable to predict that white matter pathways linking the left PFC, and in particular the LIFG, to these left temporal regions may be also involved in semantic interference resolution in language production. This idea has been partially confirmed using Diffusion Tensor Imaging (DTI) and deterministic tractography. For example, a study by Harvey and Schnur (29) found that the degree to which the left inferior fronto-occipital fasciculus (IFOF) was damaged predicted the size of the semantic interference effect in a group of chronic left hemisphere stroke patients.

Given the proposed central role of the LIFG in proactive interference resolution in working memory and the engagement of temporal and occipital brain regions in other aspects of working memory, memory formation, and recollection, it seems logical that white matter pathways linking lateral PFC regions, particularly the LIFG, to posterior cortical regions should also play a critical role in resolving proactive interference in working memory tasks. White matter pathways passing through the extreme/external capsules would be particularly well suited to carry out this function as this region constitutes a bottleneck through which ventral stream fibers, including the IFOF pass (30). Another white matter pathway known to link the IFG to the temporal lobe is the arcuate fasciculus (AF). The left arcuate fasciculus is thought to play a predominant role in various aspects of language processing, including syntactic processing (31, 32), which has been suggested to rely on working memory abilities [e.g., (33)]. Thus, the AF could also play an important role in resolving proactive interference resolution across modalities.

In the current study, we investigated the role of both gray and white matter regions in resolving proactive interference in working memory using the classic Recent Probes Test (6) in a group of unilateral stroke patients. Based on previous findings in the literature, we focused our analyses on the left and right PFC, left temporal cortex, the extreme/external capsule, the IFOF, and the arcuate fasciculi (AF). We predicted that patients with left PFC lesions would be more impaired overall on the RPT and that they would show a disproportionate proactive interference effect, relative to other patient groups. We also predicted that damage to ventral white matter pathways passing through the extreme/external capsule, including the IFOF, would positively correlate with the size of the proactive interference effect on the RPT working memory task.

## Methods

### Participants

The study was performed in agreement with the Declaration of Helsinki and was approved by the Veterans Affairs Institutional Review Board for Protection of Human Subjects. All subjects provided informed consent prior to participating.

Fifteen unilateral stroke patients (7 patients with left frontal lesions, 6 patients with left temporal lesions, and 2 patients with right frontal lesions) were evaluated for the current study on the Recent Probes Test. All patients were tested in the chronic phase of stroke (> 6 months post-onset), were pre-morbidly right-handed, and were native-proficiency English speakers, with no other prior neurologic or psychiatric history. All patients were within-normal-limits on language testing, except for one patient who had impaired speech fluency. All patients were easily able to understand and comply with task instructions.

For comparison, we also collected RPT data from 16 age- and education-matched controls (11 females). Patients and controls did not differ in respect to age [t(22.16) = −1.25, *p* = 0.224, mean age patients: 61, SD = 11 years, mean age controls: 65, SD = 6 years] or years of education [t(27.81) = 1.03, *p* = 0.313, mean years of education patients: 17 years, SD = 2 years, mean years of education controls: 16 years, SD = 2 years] see Table 1.

**Table 1:** Demographics, lesion site information, and brain imaging availability.

**Patient Number**	**Age (years)**	**Years of Education**	**Sex**	**Lesion Site**	**Lesion Volume (cm^**3**^)**	**Diffusion imaging measures**
P1	69	16	M	LT	2.393	Yes
P2	63	17	F	LF	95.186	Yes
P3	50	14	M	LF	137.237	No
P5	77	15	F	LF	93.979	Yes
P6	66	20	M	LT	18.189	No
P7	73	18	M	RF	110.246	Yes
P8	66	14	F	RF	201.512	No
P9	56	20	M	LF	107.160	No
P10	55	20	F	LF	114.054	Yes
P11	52	18	M	LF	111.452	Yes
P12	73	18	F	LT	42.950	Yes
P13	54	16	M	LT	127.807	Yes
P14	40	14	M	LF	127.283	Yes
P15	49	16	M	LT	66.000	Yes
P16	72	18	M	LT	87.771	No

*LT, Left temporal; LF, Left frontal; RF, Right frontal*.

**Table 2:** Median error rates (percentages) and inter-quantile ranges (in brackets, 25–75%) for all participant groups for the 4 trial types of the recent probes test and for the interference effect.

	**Trial type**				
	**Non-recent**		**Recent**		**Interference Effect**
**Participant group**	**Positive**	**Negative**	**Positive**	**Negative**	
Controls	8.3 (6.7-16.7)	0 (0-3.3)	3.3 (2.5-6.7)	5 (3.3-7.5)	3.3 (0-6.7)
Left frontals	26.7 (16.7-31.7)	6.7 (6.7-13.3)	23.3 (13.3-33.3)	16.7 (10-25)	6.7 (3.3-20)
Left temporals	23.3 (10-31.7)	4.2 (3.3-13.8)	15 (8.3-19.2)	16.7 (11.7-26.7)	10 (5-16.7)
Right frontals	26.7 (16.7-36.7)	5 (4.2-5.8)	6.7 (3.3-10)	16.7 (11.7-21.7)	11.7 (0-23.3)

### Materials and Design

The stimuli in the RPT were 21 English consonants, presented in lowercase for the target set and uppercase for the probe set, to avoid visual similarity between sets. Target-set letters were presented 4 at a time around a central fixation square. Target-set stimuli were presented in black Arial font, size 48. Probe letters were presented one at a time centrally (replacing the fixation square). Probe stimuli were presented in black Times New Roman font, size 64, to further avoid visual similarity with the target-set letters (see Figure 1).

There were 4 experimental conditions (probe types): (1) Recent Negatives (RN) were trials in which the probe letter was *not* part of the current target set, but was presented in the target set on the previous trial (trial *n*-1); (2) Non-recent Negatives (NN) were trials in which the probe letter was *not* part of the target set for the current trial, nor had it been presented on the previous trial; (3) Recent Positives (RP) were trials in which the probe was present in the target set on both the current (n) and previous (n-1) trial; and (4) Non-recent Positives (NP) were trials in which the probe was present in the target set on the current trial but *not* in the previous one. These four trial types (RN, NN, RP, and NP) were balanced, such that there were 30 trials of each type, 10 in each experimental block, after excluding the 2 first trials in each block as these were more prone to noisy responses.

The dependent variable on the RPT was accuracy across the four different trial types. Due to the presence of hemiparesis in most of the patients, reaction time was not considered a reliable measure and thus was not analyzed.

### Procedure

The trial sequence was as follows: (1) a warning tone (soft beep) was presented concurrently with a fixation square (1 cm x 1 cm) for 500 ms, (2) 4 lowercase target-set letters (one in each quadrant) and a smaller fixation square (0.5 cm x 0.5 cm) were presented for 2000 ms, (3) a blank screen (retention interval) was presented for 2000 ms, (4) a single uppercase probe letter was presented centrally (replacing the fixation stimulus) until the participant made a response, and (5) a blank screen was presented for 1000 ms (inter-trial interval). Participants were asked to press the left button on a computer mouse if the probe had been present in the target set and the right button, if not. Participants were tested individually in a noise-attenuated testing room on a desktop PC. The task was programmed with Neurobehavioral Systems Presentation software.

Participants were first given a series of demonstration trials that provided feedback on response accuracy. Participants then completed a practice block of minimum 9 trials with no feedback. Participants had to achieve >70% accuracy on the practice block in order to advance to the test blocks. The number of trials in the practice block was increased as necessary to achieve this criterion. For the main experiment, participants completed 3 test-blocks of 42 trials each, taking breaks in between blocks.

Participants were allowed to choose which hand to use, which, for the patients, was likely impacted by both their pre-morbid handedness and current degree of hemiparesis. Since we did not analyze reaction time data (only accuracy), the effect of hand was assumed to be negligible.

### Brain Imaging

**Figure 2:**
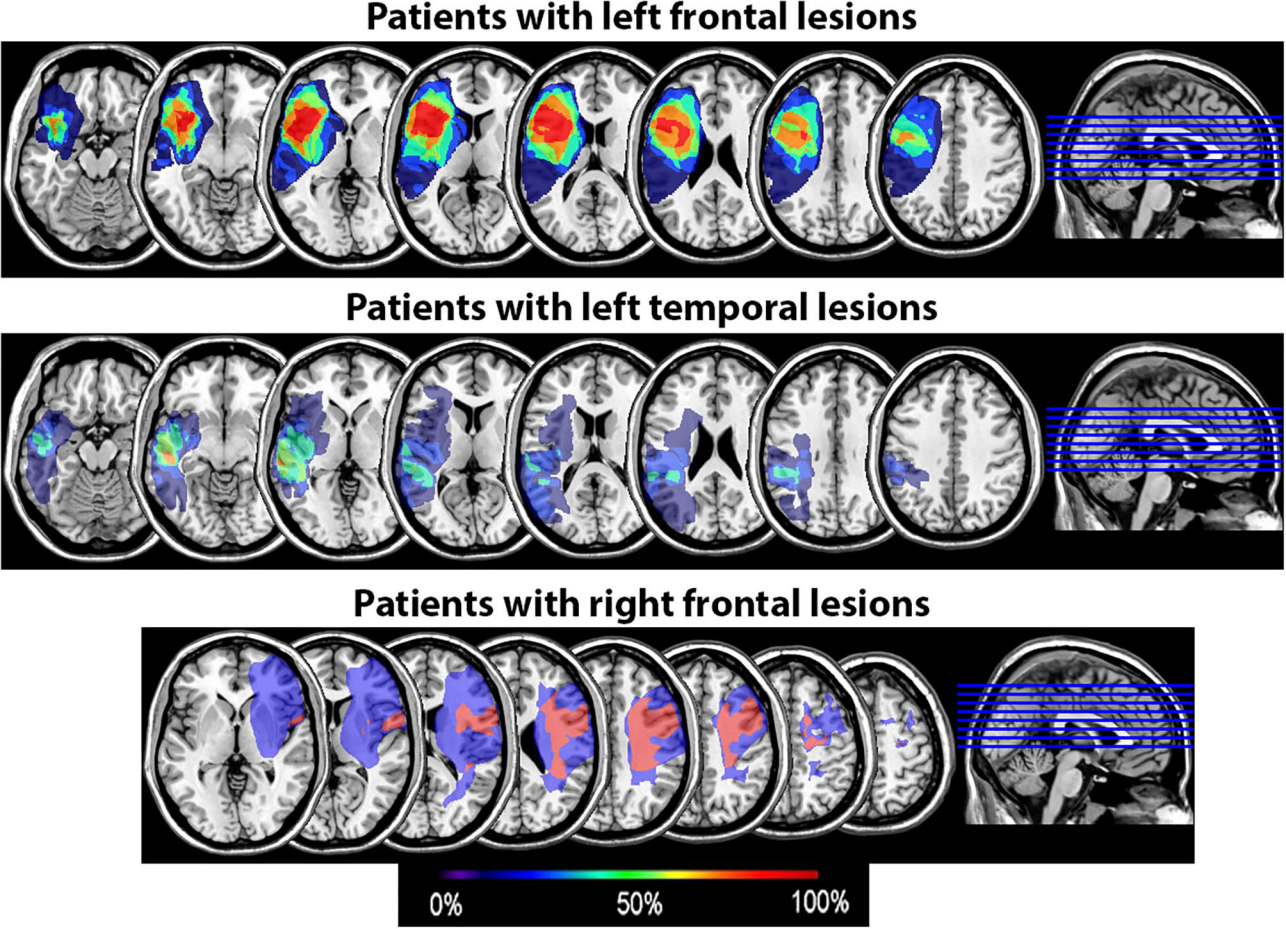
Lesion overlay of the 15 patients by group (patients with left frontal lesions, *n* = 7; patients with left temporal lesions, *n* = 6; patients with right frontal lesions, *n* = 2). The color coding indicates the amount of overlap between the different patients' lesions (red corresponds to 100% overlap and purple to 0% overlap).

All underwent structural neuroimaging on a Siemens Magnetom Verio open-bore 3T MRI scanner and were scanned and tested at least 6 months post-stroke. High resolution 3T MRI scans were acquired using a 3D T1-weighted (T1W) MPRAGE (magnetization-prepared rapid gradient echo) protocol with 1 mm^3^ isotropic resolution (TR/TE/TI = 2400/3.16/1000 ms, flip angle = 8°; FOV = 256 mm; imaging matrix = 256 x 256; acquisition time = 4.5 min). Two T1W images were acquired and averaged to improve the contrast-to-noise ratio. FLAIR and fast spin echo T2-weighted (T2W) images were also acquired with the default Siemens pulse sequences to improve the visual assessment of brain lesions. Diffusion tensor imaging (DTI) and resting-state fMRI sequences were also collected pre- and post-SPT intervention on a subset of patients, in order to examine any neural changes associated with SPT in future analyses. Lesioned areas were delineated in MRIcron (34) by a trained researcher with verification by a neurologist using input from T1, T2, and Flair scans or, in one case, from a CT scan when no MRI was available. Lesion overlays showing the extent of lesion overlap in each patient group are shown in Figure 2.

High angular resolution diffusion imaging (HARDI) was acquired in 11 of the 15 patients on the same scanner with a 12-channel head coil. Single-shot spin echo-planar imaging (EPI) was performed. Each slice was 2 mm^3^ thick (bandwidth = 1812 Hz). A total of 65 axial slices were imaged in the inter-commissural plane (interleaved, no gap) for whole-cerebral coverage (voxel size = 2 x 2 x 2mm^3^, flip angle = 90; GRAPPA acceleration factor = 2, 6/8 partial Fourier sampling; field mapping for off-line EPI distortion correction; TR/TE = 17600/93.6 ms). Sixty four isotropic diffusion directions were applied at b = 2000 s/mm^2^ with 10 b = 0 images as reference, and field maps were acquired to counteract EPI spatial distortion. The 3D T1 weighted-images were acquired using an MP-RAGE (magnetization-prepared rapid gradient echo) protocol with 1 mm^3^ isotropic resolution (TR/TE= 2200/1.62 ms, flip angle = 8°; FOV = 256 mm; 192 sagittal slices imaging matrix = 256 x 256). The T2 weighted images were acquired with the same imaging resolution, but the TR/TE was 3000/409 ms). Two-fold GRAPPA accelerated imaging on a 12 channel head coil was used for both T1 and T2-weighted imaging. Total scanning time was 22.5 min.

### Data Analysis

#### Behavioral Data

Statistical analyses were performed using “R” statistical software [v 3.1.1 (35)]. The “lmerTest” package was used to compute the mixed effect model (36) and “car” was used to compute deviance tables for the fixed effects (37). Accuracy on the RPT was analyzed with a generalized logistic mixed-effects model (38, 39), which relies on single-trial data rather than on averages over participants or items. This model is free from the assumptions of homogenous variance and sphericity (40). The main effect of Recency (RN vs. NN^1^) was analyzed as a within-participant factor, and Study Group (controls, left PFC, left temporal, and right frontal) was a between-participant factor. Random effects included an intercept for Participant, as well as by-participant random slopes for Recency.

#### Lesion Site Analysis

Diffusion Tensor Imaging (DTI) measures, including fractional anisotropy (FA) and mean diffusivity (MD), were computed with the MRTrix software, version 2 (5). Diffusion MRI datasets were linearly co-registered to the MPRAGE T1-weighted high-resolution (1 mm isotropic voxels) anatomical image using the boundary-based registration technique implemented in FSL software package FLIRT_BBR (https://fsl.fmrib.ox.ac.uk/fsl/fslwiki/FLIRT_BBR). The T1-weighted images were normalized to MNI 152 space using the unified segmentation normalization (41) in SPM8. Manually reconstructed lesion masks were used for cost-function masking (42). The spatial transformation obtained from the normalization of the T1-weighted anatomical image were applied to the DTI maps so that they could be analyzed in relation to the probabilistic maps provided in the Johns Hopkins University DTI-based white-matter atlas (43) for the arcuate fasciculus (AF), Inferior Fronto-Occipital Fasciculus (IFO), and extreme/external capsule (EC). To quantify tract integrity, the threshold for the probabilistic tract maps was set at 50% probability. FA and diffusivity maps were sampled at and averaged over the *p* > 50% sections of each tract. The T1 and T2 images were corrected for spatial biases due to MRI scanned magnetic field inhomogenity, and intensity normalized to correct for MRI signal differences across scanning sessions. The mean non-diffusion weighted (B0) image was linearly co-registered with the T2-weighted image. In sum, FA, MD, T1, and T2-weighted images were used to assess degree of damage in each fiber tract in the ipsilesional hemisphere.

Correlations between the normalized degree of damage in a given fiber tract ipsilateral and the normalized size of the interference effect (as defined by the error rate in the RN trials minus the error rate in the NN trials) were assessed using non-parametric Spearman rank correlation tests. Normalization was performed by taking the value of interest (interference effect, FA, MD, T1, or T2 measure) per patient, subtracting the average value over all participants, and dividing by the standard deviation around the mean. FA, MD, T1, or T2 values per tract were always taken in the ipsilateral hemisphere relative to the lesion site. Resulting *p*-values were Bonferroni-corrected for multiple comparisons per measure (FA, MD, T1, and T2). We also controlled for the potential confound of lesion size and computed follow-up linear regressions with lesion size as a factor.

In addition, in order to examine whether white matter fiber tract damage provided a more informative factor than cortical lesion site, we re-grouped the patients depending on the amount of damage in the three fiber territories: IFOF, EC, and AF. We calculated the median of the normalized T2-weighted signal for each of these structures and binned the patients depending on whether or not they had a higher or lower normalized T2-weighted signal than the median in each of these structures. Since there were 11 patients with available data for this analysis, there was an unequal number of patients in each group. We compared the groups using non-parametric Mann-Whitney U tests for independent samples.

For one patient (P13), we performed tractography using the MRTrix software package version 3 (5). Probabilistic streamline tractography with the iFOD2 algorithm was employed to generate 5,000,000 streamlines, seeded randomly from a whole brain mask and varying in length from 10 to 200 mm. Default options in MRTrix were used, with a fiber orientation distribution (FOD) amplitude of 0.1 and an angle threshold of 45 degrees for streamline termination. For visualization with the TrackVis software package (www.trackvis.org), 250,000 streamlines varying in length from 50 to 150 mm were selected. Regions of interest (ROI) for delineating the AF and IFOF were manually drawn on the patient's T1-weighted anatomical image as described in Zhang et al. (44). Streamlines passing through these areas were selected to visualize the AF and the IFOF.

## Results

### Behavioral Performance

**Figure 3:**
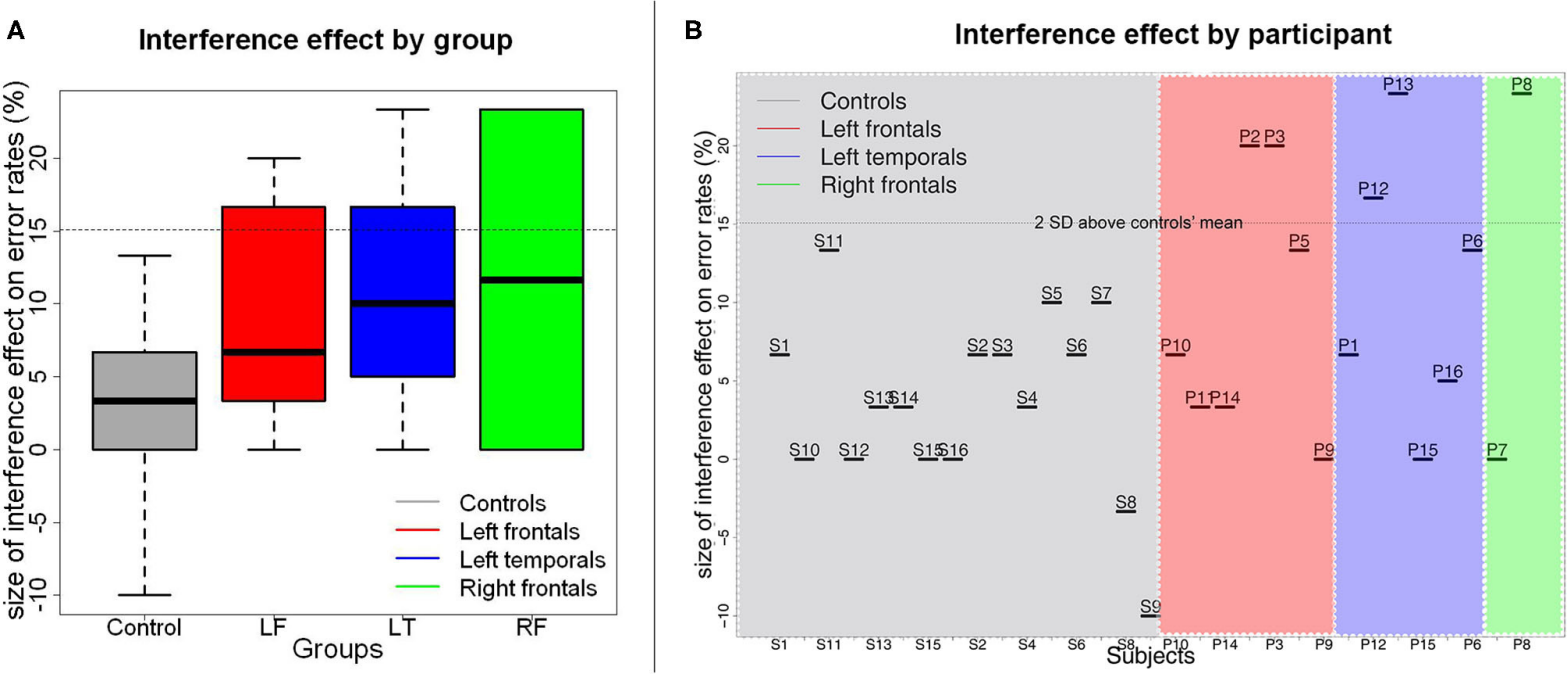
Size of the proactive interference effect on error rates by patient group **(A)** and by participant **(B)**. In **(A)**, medians are indicated by the black horizontal lines in the box-and-whisker plots. Interquartile ranges are represented by the boxes and the total range is depicted by the dotted lines. The horizontal dotted line indicates the value corresponding to the controls' mean interference effect size plus 2 standard deviations above the controls' mean. As can be seen in **(B)**, a few participants from each patient group had interference effects that were larger than this value.

There was a significant effect of Recency on accuracy rates (*Wald* *χ*^2^(1) = 21.13, *p* < 0.001). As predicted, participants were overall less accurate in the RN condition than in the NN condition (*β*_*raw*_ = −6.15 × 10^−1^, *CI*= [-8.91 × 10^−1^−3.39 × 10^−1^], *SE*= 1.41 × 10^−1^, *Z*=-4.37, *p* < 0.001). There was also a significant effect of Group on accuracy (*Wald χ*^2^(3) = 28.10, *p* < 0.001), indicating that patients with left frontal lesions performed less accurately than controls (*β*_*raw*_ = −5.44 × 10^−1^, *CI*= [-11.06 × 10^−1^,1.85 × 10^−2^], *SE*= 2.87 × 10^−1^, *Z* = −1.90, *p* = 0.058). No other between group comparisons were significant (patients with left temporal lesions vs. controls: *β*_*raw*_ = −4.88 × 10^−1^, *CI*= [-10.82 × 10^−1^ 1.06 × 10^−1^], *SE*= 3.03 × 10^−1^, *Z* = −1.61, *p* = 0.107; patients with right frontal lesions vs. controls: *β*_*raw*_ = −1.16 × 10^−1^, *CI*= [-10.19 × 10^−1^ 7.86 × 10^−1^], *SE* = 4.60 × 10^−1^, *Z* = −0.253, *p* = 0.800). There was no interaction between Recency and Group [*Wald χ*^2^(3) = 0.63, *p* = 0.889, see Figure 3A).

#### Correlations With White Matter Damage

**Figure 4:**
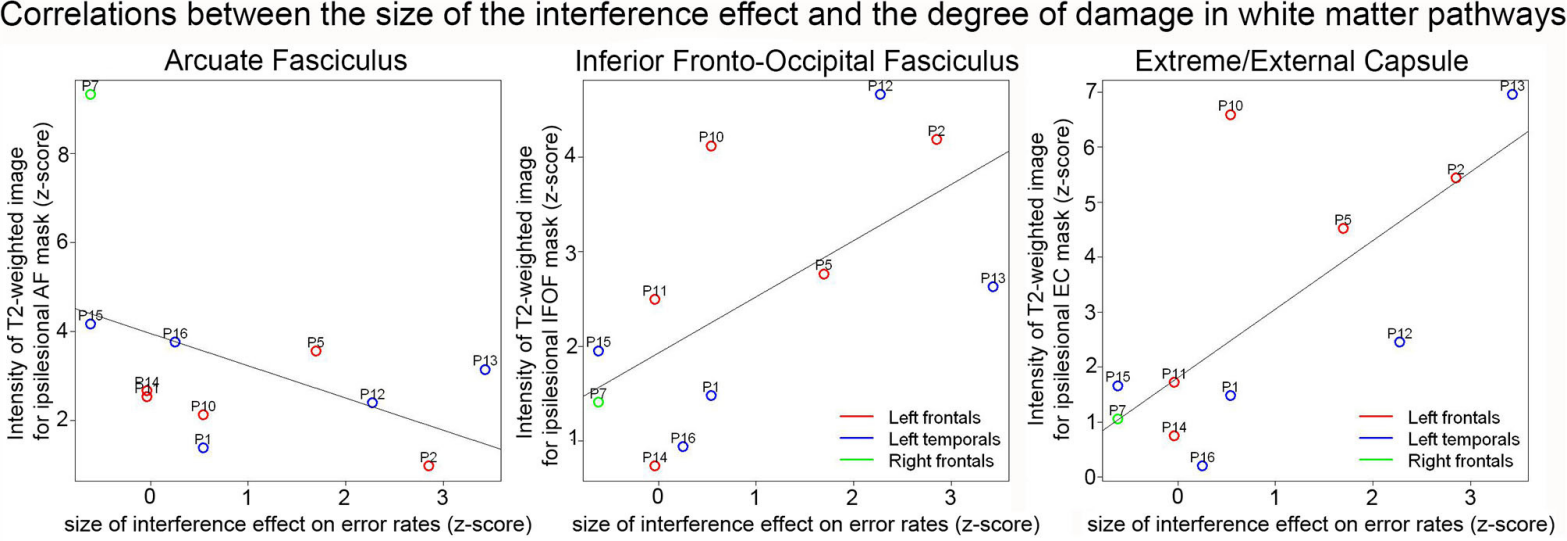
Correlation plots between the normalized size of the interference effect on error rates and the normalized intensity of the T2-weighted image for the ipsilesional arcuate fasciculus (left), IFOF (middle), and extreme/external capsules (right). The color of the dots indicates the patient groups.

Correlations between RPT performance and white matter damage on z-scored values are presented in Table 3. Significant positive correlations were found between the size of the interference effect and the T2-weighted signal in the Extreme/External capsule (EC) (S = 62.93, p_corr_ = 0.042, rho = 0.714). The positive correlation between the size of the interference effect and the T2-weighted signal in the inferior fronto-occipital fasciculus (IFOF) was marginal after correction for multiple comparisons (S = 68.97, p_corr_ = 0.060, rho = 0.687) (see Figure 4). No other comparisons were significant. In particular, there was no significant correlation between the T2-weighted signal in the arcuate fasciculus (AF) and the size of the interference effect (S = 343.85, p_corr_ = 0.231, rho = -0.563).

**Table 3:** Spearman rank correlation test results between the normalized (z-score) interference effect per patient and the normalized (z-score) amount of signal per fiber tract and by measure (Fractional Anisotropy, FA; Mean Diffusivity, MD; T1-weighted signal, T1; T2-weighted signal, T2, for the ipsilesional masks of the white matter territories of interest).

	**Measure**			
**Fiber tract territory**	**FA**	**MD**	**T1**	**T2**
Arcuate fasciculus (AF)	S = 125.93	S = 306.07	S = 138.44	S = 343.85
	rho = 0.428	rho = −0.391	rho = 0.371	rho = −0.563
	*p*_corr_ = 0.570	*p*_corr_ = 0.702	*p*_corr_ = 0.786	*p*_corr_ = 0.231
Inferior fronto-occipital fasciculus (IFOF)	S = 314.37	S = 122.33	S = 288.47	**S = 68.97**
	rho = −0.429	rho = 0.444	rho = −0.311	**rho = 0.687**
	*p*_corr_ = 0.564	*p*_corr_ = 0.513	*p*_corr_ = 0.999	**p**_**corr**_ **=** **0.060**
Extreme/external capsule (EC)	S = 288.12	S = 132.71	S = 324.96	**S = 62.93**
	rho = −0.310	rho = 0.397	rho = −0.477	**rho = 0.714**
	p_*corr*_ = 1	*p*_corr_ = 0.681	*p*_corr_ = 0.414	***p***_**corr**_ **=** **0.042**

*Significant and marginally significant (p < 0.10) correlations are in bold. P-values were Bonferroni-corrected for multiple comparisons per measure*.

To control for the potential confound of lesion size, we computed linear regressions with lesion size as a factor. Lesion size had no significant impact on the size of the interference effect, and the significant effect of ventral white matter damage remained significant (see Supplementary Table 1 for statistical details).

Next, we analyzed the size of the RPT interference effect based on the amount of damage in the three fiber territories. Patients with a greater degree of IFOF damage (*n* = 5) had a larger interference effect than patients with a T2-weighted signal below the median (*n* = 6) (W = 29.5, *p* = 0.010). This effect was also observed for the EC (W = 27, *p* = 0.035; *n* = 6 for high, *n* = 5 for low). There was no significant effect of the degree of damage in the AF (W = 12, *p* = 0.646; *n* = 5 for high, *n* = 6 for low).

#### Single-Case With Preserved LIFG and Large Interference Effect

**Figure 5:**
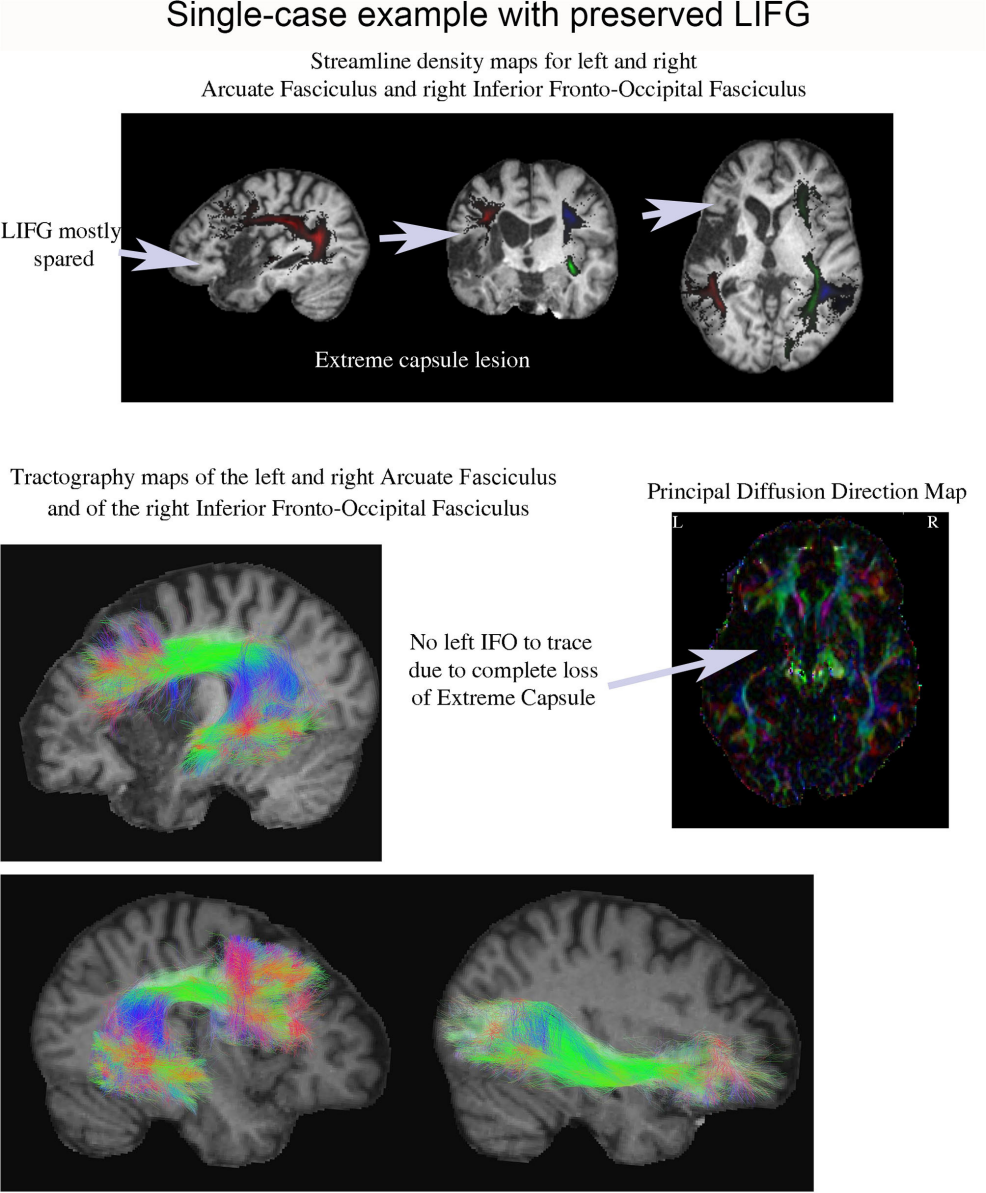
Streamline density and tractography maps for the left and right arcuate fasciculus and the right IFOF in P13 (who had one of the largest proactive interference effects), and the principal diffusion direction map. This patient's lesion was centered in the anterior left temporal lobe and his LIFG was mostly spared. Remarkably, it was impossible to tract his left IFOF suggesting it was severely damaged.

One patient (P13) was of particular interest because he exhibited a very large interference effect (> 3 SDs above control mean), but his LIFG was mostly spared (see Figures 3–5). Tractography in this patient showed complete loss of the extreme/external capsule and nearly complete absence IFOF (Figure 5).

## Discussion

In the current study, we assessed proactive interference in working memory using the Recent Probes Test in a group of chronic stroke patients with lesions in the left or right PFC, or left temporal cortex. Contrary to prediction, patients with left PFC lesions did not show a reliably greater interference effect than patients with right PFC lesions, patients with left temporal lesions, or controls. In contrast, the amount of damage in the extreme/external capsule did positively correlate with the size of the interference effect. There was also a marginal positive correlation between the amount of damage in the IFOF and the size of the interference effect. This suggests that ventral white matter pathways passing through the extreme/external capsule and connecting the LIFG to left posterior regions play a critical role in resolving proactive interference in working memory.

### Role of Cortical Lesion Site in Working Memory

Prior studies have found that the bilateral PFC plays a key role in working memory (45–47). Indeed, patients with left or right lateral PFC lesions have been found to be impaired in delayed match-to-sample tasks, especially when distractors are presented during the delay period [(48) and see (49) for a review]. In the current study, using a verbal version (with letters as stimuli) of the recent probes paradigm and no intervening distractors, we found that only the patients with left PFC lesions were less accurate than the controls. The present study therefore supports the idea that the lateral PFC plays an important role in working memory but further suggests the that the left PFC is more critical than the right PFC in supporting working memory when verbal stimuli are used. Moreover, it appears that this is the case even without the use of distractors between the target and the probe. Our results are therefore partly consistent with those of Thompson-Schill et al. (12), who also used verbal stimuli in the RPT and found impairments in baseline memory performance in patients whose brain damage was primarily circumscribed to the left PFC^2^. This suggests that the verbal version of the recent probes paradigm does elicit left-PFC-dependent working memory abilities. Differences between the current study and those reviewed by D'Esposito and Postle (49) are that most of the prior studies involved heterogeneous patient groups and non-verbal stimuli, which were visually similar between the target-set and the probe. In the current study, patients were separately classified as having lesions in the left PFC, right PFC, or left temporal lobe. Moreover, the stimuli were visually dissimilar between the target set and the probe (i.e., lower case for the target-set and upper case for the probe), which may explain the fact that working memory impairments were observed despite of the absence of distractors. Together this suggests the left lateral PFC may be more critical than the right PFC in supporting working memory for the letter stimuli in this particular paradigm. This is consistent with the fact that verbal working memory components have generally been found to be left lateralized [e.g., (50, 51)]. We note however that there were only 2 patients with right frontal involvement in our study and that the size of the interference effect was very different between these two patients. Further investigation is therefore needed to confirm the relative role of the right vs. left PFC in the resolution of interference in working memory.

### Neuroanatomical Basis of Proactive Interference Resolution

Our proactive interference results did not replicate prior results showing that damage to the LIFG results in greater proactive interference effects, as reported by Thompson-Schill et al. (12) using the RPT. Indeed, we did not observe an interaction between Group and Recency even though most patients with left PFC lesions in our group had lesions encompassing at least the posterior portion of the LIFG (Figure 2 and Supplementary Figure 1). It has been suggested that it is specifically the pars triangularis (BA 45) that is critical in resolving proactive interference (7). In our patient group however, at least three patients (P10, P11, and P14) had lesions encompassing BA45 but still had an interference effect within normal range (Figure 3). This suggests that lesions to this part of the LIFG do not always result in increased proactive interference in the RPT as seen in accuracy rates. This further points to a high degree of variability in the precise cortical site that is associated with proactive interference resolution.

Another way to investigate the neuroanatomical basis of proactive interference resolution is to examine the underlying white matter pathways that link the lateral PFC to the temporal lobe. Here, we observed that damage to the extreme/external capsules as measured through the intensity of the T2-weighted signal was a stronger predictor of the size of the interference effect than cortical lesion site. In other words, damage to the pathways passing through these areas was linked to larger proactive interference effects. This suggests that these white matter territories contribute significantly to supporting proactive interference resolution ability. There was also a marginal positive correlation between the size of the interference effect and the degree of damage in the IFOF territory. We note that several white matter pathways are known to pass through the extreme/external capsules including the IFOF as well as the Uncinate Fasciculus (30). The UF connects the anterior temporal lobes to the anterior PFC and orbitofrontal cortex. Though the UF has not been directly associated with proactive interference in language, it has been associated with semantic control in word comprehension (52). Thus, other white matter pathways passing through these capsules may also be involved in proactive interference resolution. The fact that the correlation coefficient was slightly higher for the extreme/external capsule measurement than for the IFOF would be in agreement with this idea. Determining which additional pathways may be involved however will require further testing.

A particularly compelling example of the importance of the ventral white matter pathways in proactive interference resolution is provided by the single case example (P13) presented in section Single-Case With Preserved LIFG and Large Interference Effect. The LIFG was largely preserved in this patient but the ventral white matter pathways linking the LIFG to the temporal lobe had been severely damaged. Remarkably, the interference effect observed in this patient was more than three standard deviations above the mean interference effect observed in the controls. This further implies that the role inferior frontal cortex in resolving interference in working memory may be heavily dependent on anatomical connections to the temporal lobe through the ventral white matter pathways.

The fact that ventral white matter pathways may be involved in supporting proactive interference resolution in the RPT suggests that similar pathways may underly proactive interference resolution in both language production and working memory when verbal stimuli are used. Indeed, as previously mentioned, damage to the left IFOF has been shown to be positively correlated with the size of the semantic interference effect in language production (29). In addition, damage to the Uncinate Fasciculus has been shown to be positively correlated with semantic control impairment in word comprehension (29). The semantic interference effect is caused by residual activation of semantically-related representations in memory and has been considered to be analogous to the proactive interference effect in working memory (7, 18, 19). As reviewed in the introduction, interference resolution in both cognitive domains has implicated the LIFG (8, 9, 12, 25). Here, we suggest that these types of interference resolution may also rely on the same neural circuitry. Thus, this study provides some of the first evidence for the involvement of a common neuroanatomical white matter pathway network for resolving proactive interference in both language production and working memory when verbal stimuli are used.

Although we did not observe significant correlations between the size of the interference effect and the degree of damage in the white matter structures under study using the other white matter measures (i.e., fractional anisotropy, mean diffusivity, and T1-weighted anatomical masks), there are plausible explanations. One reason may lie in the fact that FA and MD measures may be more sensitive to noise. Indeed, T2 signal has higher spatial resolution than diffusion imaging showing fluid-filled lesion cavities very clearly, and has been used as an indicator of brain damage in the other studies [e.g., (53, 54)]. A second reason is that this study was limited in the overall number of patients who had DTI data available for analysis. Thus, while our findings that ventral white matter pathways are likely involved in proactive interference resolution are timely and ties together many prior findings within in the literature, determining the precise nature of the most critical pathways remain exploratory and will need to be confirmed by in future studies.

The findings reported here regarding the importance of ventral white matter pathways in working memory and proactive interference resolution are important both theoretically and clinically. The observation that damage to white matter pathways was a stronger predictor of performance than damage to cortical lesion sites is consistent with language studies that have focused on aphasia [e.g., (55–59)], and also in awake language mapping studies in tumor resection cases [e.g., (60, 61)]. For example, it has been observed that damage to subcortical structures and white matter pathways beneath Broca's area is a stronger predictor of Broca's aphasia than damage to Broca's area itself (55). This points to a greater degree of plasticity of the inferior frontal gyrus in comparison to the underlying white matter pathways, as has been suggested for cortical regions in comparison to white matter pathways in general (61). Indeed, patients with lesions restricted to Broca's area (i.e., the LIFG) often only show transient marked language production deficits, which resolve into milder types of aphasia within 3 to 6 months, such as transcortical motor or anomic aphasia (62). The consequences are much worse, however, if the underlying white matter pathways that normally connect the LIFG to the rest of the brain are damaged (57).

In summary, the LIFG may not be the only critical structure needed to support proactive resolution in working memory. In fact, it appears that other cortical structures may be involved in supporting this function when the LIFG is damaged. The findings reported here instead highlight the role that ventral white matter pathways passing through the extreme/external capsule play in resolving proactive interference in working memory, by connecting the LIFG to other areas within the left posterior cortex. Theoretically, the findings reported here also provides some of the first evidence in support of a common neuroanatomical network for resolving proactive interference in both language production and working memory.

## Data Availability Statement

The raw data supporting the conclusions of this article will be made available by the authors, without undue reservation.

## Ethics Statement

The studies involving human participants were reviewed and approved by VA Northern California Healthcare System Institutional Review Board. The patients/participants provided their written informed consent to participate in this study.

## Author Contributions

AT, SR, and KS conceived and planned the study. ND provided patient access and infrastructure support. SR and KS carried out the experiment. SR, TH, and AT planned and performed the data processing and analyses. SR, KS, TH, JB, ND, and AT contributed to the interpretation of the results. SR took the lead in writing the manuscript. KS, JB, ND, and AT provided critical feedback and helped shape the research, analysis and manuscript. All authors contributed to the article and approved the submitted version.

## Conflict of Interest

The authors declare that the research was conducted in the absence of any commercial or financial relationships that could be construed as a potential conflict of interest.

## Footnotes

^1^We focused on the RN vs. NN comparison as we were primarily interested in the interference effect. However, we report the median error rates and inter-quartile ranges for all conditions in Table 2.

^2^All but one patient in Thompson-Schill et al. (12) had left PFC lesions. See below for differences between our study and the Thompson-Schill et al. (12) study regarding proactive interference.

## Supplementary Material

Supplementary Figure 1 Lesions from individual patients with left frontal lesions in red. All were reconstructed in the individual patient's native scan space and are presented over each patient's T1 MRI image except for P3 for whom only a CT scan was available. This patient's lesion is presented on the MNI template.
