# Supplementary figures and images for: Neural Underpinnings of Proactive Interference in Working Memory: Evidence From Patients With Unilateral Lesions
Source: Front Neurol. 2021 Feb 10;12:607273. doi: 10.3389/fneur.2021.607273 (PMC7902939; doi:10.3389/fneur.2021.607273)

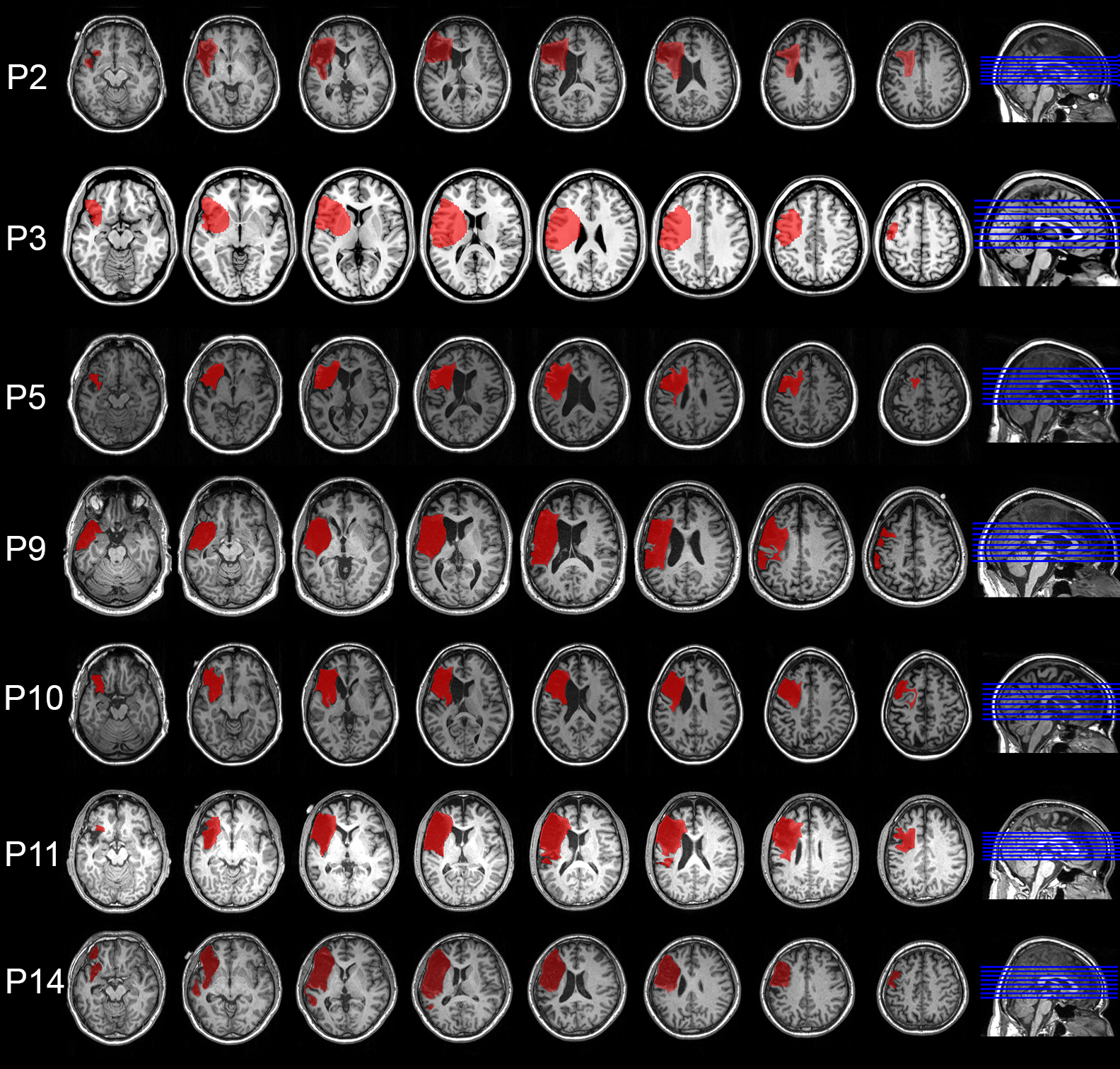

Supplement: Supplementary Figure 1 — Lesions from individual patients with left frontal lesions in red. All were reconstructed in the individual patient's native scan space and are presented over each patient's T1 MRI image except for P3 for whom only a CT scan was available. This patient's lesion is presented on the MNI template. [file Image_1.TIF]
