# Supplementary material for: Neural Underpinnings of Proactive Interference in Working Memory: Evidence From Patients With Unilateral Lesions
Source: Front Neurol. 2021 Feb 10;12:607273. doi: 10.3389/fneur.2021.607273 (PMC7902939; doi:10.3389/fneur.2021.607273)
Supplement: Supplementary file 2 [file Table_1.pdf]

Table S1: Results of the linear regressions performed on the size of the interference effect per white matter tract territory (as measured by the intensity of the T2-weighted signal in the mask of interest) and including lesion size as a factor. Significant and marginal effects are in bold.

	Fiber tract territory		
	Arcuate fasciculus (AF)	*Inferior fronto-occipital fasciculus (IFOF)*	*Extreme/external capsule (ExC)*
Intercept	$\beta = 2.63 \times 10^{-1}$<br>SE = 1.32×10^{-1}<br>t-value = 2.00<br>p = .081	$\beta = -3.10 \times 10^{-1}$<br>SE = 1.84×10^{-1}<br>t-value = -1.69<br>p = .131	$\beta = -1.45 \times 10^{-1}$<br>SE = 8.63<br>t-value = -1.68<br>p = .132
Intensity of T2-weighted signal in white matter tract territory	$\beta = -1.24 \times 10^{-1}$<br>SE = 7.93×10^{-2}<br>t-value = -1.56<br>p = .157	**$\beta = 2.52 \times 10^{-1}$**<br>**SE = 1.13×10^{-1}**<br>**t-value = 2.42**<br>**p = .055**	**$\beta = 1.59 \times 10^{-1}$**<br>**SE = 5.03×10^{-2}**<br>**t-value = 3.16**<br>**p = .013**
Lesion size	$\beta = 3.55 \times 10^{-5}$<br>SE = 6.76×10^{-5}<br>t-value = 0.56<br>p = .614	$\beta = 1.52 \times 10^{-5}$<br>SE = 5.85×10^{-5}<br>t-value = 0.26<br>p = .801	$\beta = -4.56 \times 10^{-5}$<br>SE = 5.25×10^{-5}<br>t-value = -0.87<br>p = .410
